# Impact of group dancing during Japanese festivals on people’s sense of community

**DOI:** 10.3389/fpsyg.2025.1469066

**Published:** 2025-03-24

**Authors:** Satoshi Kawase, Kei Eguchi

**Affiliations:** ^1^Faculty of Psychology, Kobe Gakuin University, Kobe, Japan; ^2^Independent Researcher, Osaka, Japan

**Keywords:** dance, music, festival, embodiment, social bonding, sense of community, isolation, loneliness

## Abstract

Moving together or attending festivals has been reported to foster social bonding. However, whether festivals with and without dancing affect individuals’ social bonds and sense of community remains unclear. The existing research does not demonstrate whether lasting effects exist over time, even when community festivals are held only a limited number of times a year. To address this issue, this study examines the impact of dancing at local festivals on individuals’ sense of community. This study hypothesized that if dancing with others enhances social bonding, individuals’ participation in festivals may enhance sense of community since dancing is a part of many festivals. Accordingly, an online survey was conducted a few months after a community festival, and participants responded to three scales: the Brief Sense of Community Scale, Community Consciousness Scale, and UCLA Loneliness Scale. The results found (1) that the participants who attended and danced at a festival with dancing showed a higher sense of community and lower loneliness level than those who did not dance or those who attended a festival without dancing. (2) Previous festival attendance habits did not influence these tendencies. (3) Furthermore, these tendencies were not related to the individual’s willingness to attend festivals. Therefore, dancing at festivals may promote a greater sense of community than attending festivals without dance.

## 1 Introduction

Moving the body in response to music is considered an enjoyable experience worldwide (Fitch, 2015; Stevens, 2012). A typical example of people moving their bodies in accordance with music occurs during festivals. Various types of traditional dances are performed at festivals around the world (Frederiksen and Chang, 2023). As shown in the UNESCO List of Cultural Heritage, dance is performed at ceremonies and festivals all over the world (United Nations Educational, Scientific and Cultural Organization, 2024). For example, Hula in Hawaii, Bon Odori (Bon dance) in Japan, Irish Stepdance in Ireland, Ghoomar in India, Maypole Dance in the United Kingdom, and Samba in Brazil. These dances reflect the cultural heritage and traditions of their respective regions, often performed during festivals or special occasions. Argan et al. (2021) showed that a Turkish dance festival could offer benefits, serving as an important experiential space that could improve the physical and social confidence of participants and strengthen intergenerational solidarity. Argan et al. (2021) also demonstrated that participants at the Turkish dance festival demonstrated a significant relationship between the motivation to gain dance experience and the quality of the experience; furthermore, this quality significantly impacted satisfaction. This finding suggests that the intention to participate in the festival may affect the participants’ sense of community. Furthermore, community-based festivals and community dances have been associated with a sense of community (Kino, 2016; Shinonaga et al., 2020).

Regarding the benefits of dancing and group dancing, many studies have shown that dance is a beneficial activity that promotes physical, psychological, and social health in various age groups (Fong Yan et al., 2024; McCrary et al., 2021). In a review article, McCrary et al. (2021) demonstrated the physiological, cognitive, and psychological benefits of dance. They indicated that dance functions as an aerobic exercise that improves cardiorespiratory endurance, increases muscle strength and flexibility, and improves balance and physical coordination. Furthermore, they also pointed out that learning and remembering choreography helps to maintain and improve cognitive function. As for psychological effects, dance promotes the secretion of endorphins, reduces stress and anxiety, and enhances mood. As a social benefit, group dance activities can strengthen social ties, promote social interaction, reduce feelings of isolation, and contribute to a sense of community (McCrary et al., 2021). Dancing together can also promote the secretion of oxytocin and strengthen social bonds (Vander Elst et al., 2023). The benefits of dance have also spread to dance movement therapy (DMT) and dance interventions in medical institutions. DMT is said to be effective for reducing depression and anxiety, improving quality of life, and improving interpersonal, cognitive, and motor skills (Koch et al., 2014). Dance is also regarded as a promising treatment and rehabilitation method for neurodegenerative diseases such as Parkinson’s disease and Alzheimer’s disease (Vander Elst et al., 2023).

In many traditional dances, music is essential. Music and its accompanying physical movements are cultural behaviors, and they are activities that people have long been familiar with (Trehub et al., 2015). Indeed, in concerts, people can be seen swaying their bodies (Keller and Rieger, 2009). In sports activities, audience members sing while jumping up and down to cheer athletes or teams (Knijnik, 2018). Furthermore, both music and dance are associated with festivals (Frederiksen and Chang, 2023). Extensive scientific research has investigated the physiological, cognitive, developmental, evolutionary, and social aspects of moving the body to music and its effects on relationships with others (Cirelli et al., 2014; Kirschner and Tomasello, 2010; Savage et al., 2020; Tarr et al., 2016). In recent years, the urge to move one’s body in response to music has been termed “groove” (as reviewed in Etani et al., 2024). The urge to move to music is probably a universal sensation because words describing the relationship between music and physical movements exist in many cultures (Etani et al., 2024; Kawase, 2024; Kawase and Eguchi, 2010). Regarding groove, groovy music can be the optimal music for anticipation mechanisms and activate the reward system. Anticipation and reward system are related to social connections (Fiveash et al., 2023). These studies highlight the significance of the link between rhythmic factors in music and physical movements. In addition, the fact that rhythmic physical movements are observed early during a person’s developmental stages indicates that moving (or dancing) to music is a primitive characteristic of human beings (Trehub et al., 2015).

In response to the question of why people move to music, its evolutionary and adaptive significance is often highlighted. In this respect, its role in fostering social bonds deserves special mention (Dunbar, 2011). Indeed, moving together to music promotes prosocial behavior (Tarr et al., 2016) and social bonding (Savage et al., 2020). This is true for both infants and adults and is considered a fundamental human trait (Cirelli et al., 2014; Kawase et al., 2018; Kirschner and Tomasello, 2010). According to Cirelli et al. (2014), 14-month-old infants recorded a higher level of spontaneous helping behavior toward adults who moved together to music compared with those who did not move together. In an experiment conducted by Kirschner and Tomasello (2010) on 4-year-old children, two people who played instruments together with the children who moved while playing a musical instrument together showed more willingness to help the other child in trouble compared to those who simply walked together. Further, in an experiment on high school students, dancing together was found to release endorphins, which foster social hand-bonding (Tarr et al., 2016). These studies show that the formation of social bonds through movement in synchronization with music is a universal phenomenon observed across the lifespan from infancy to adulthood. This ubiquity suggests that the link between synchronized movement, music, and social bonding may have evolutionary roots, implying that this behavior has been selectively advantageous throughout human history. According to Kawase et al. (2007), when two people who drummed freely engaged in communication, a shared rhythm was correlated with good interpersonal impressions. The effects of movement synchronization have also been explained by brain mechanisms, which may enhance social bonding because of increased neural synchrony and enhanced interpersonal coordination (as reviewed in Basso et al., 2020).

Simply moving together is known to influence a person’s prosocial behavior, as well; this is because synchronization promotes prosocial behavior (Carpenter et al., 2013; Keller et al., 2014), cooperative skills (Valdesolo et al., 2010), compassion and altruistic behavior (Valdesolo and DeSteno, 2011), empathy (Behrends et al., 2012), interpersonal likeability (Hove and Risen, 2009), rapport (Lakens and Stel, 2011), trust (Launay et al., 2013), and social closeness (Tarr et al., 2016). These effects are applicable to young children, as well. For instance, Carpenter et al. (2013) showed that 18-month-old infants were more helpful to adults who imitated the infants’ behavior than to those who did not. Nevertheless, Stupacher et al. (2017) pointed out the importance of the social bonds generated by moving to matching music over those occurring from simply matching movements or monotonous stimuli, such as a metronome.

One of the most common examples of dancing to music occurs during festivals. Indeed, festivals help maintain the sustainability and stability of communities (Dunbar, 2011). Indeed, festivals and rituals involving dancing to music are prevalent in various communities worldwide (Stevens, 2012; Trehub et al., 2015); therefore, festivals highlighting music and dance are important targets to examine the cultural and evolutionary behavior of human communities. Furthermore, earlier studies indicate that participation in festivals enhances social capital (Arcodia and Whitford, 2006; Jaeger and Mykletun, 2013). Festivals have been shown to promote a sense of community (Hassanli et al., 2021), enhance community building (Mair and Duffy, 2020), and ensure community maintenance (Black, 2016) as part of improving social sustainability. Accordingly, festivals are predicted to affect community awareness and social connectedness.

This study examines whether dancing, which is an inherent part of many festivals, is associated with individuals’ sense of community. Studies indicate that individuals’ prosocial behavior and sociability increase after dancing and playing music with others (e.g., Dunbar, 2011; Kawase et al., 2018; Keller et al., 2014; Tarr et al., 2016). Many experimental studies clarify that an individual exhibits prosocial behavior immediately after moving to music (e.g., Cirelli et al., 2014; Kirschner and Tomasello, 2010). If dancing with others enhances social bonding, individuals’ participation in festivals may enhance social bonding since dancing is a part of many festivals.

However, it is unclear whether such effects persist over time since festivals and rituals, such as community festivals and ceremonies, are held only a limited number of times (usually once) a year. In the aforementioned research, individuals’ promotion of prosocial behavior by moving to music was often measured immediately after the movement. In other words, the long-term effects of dancing associated with festivals, which occur very infrequently, remain unverified. This may be due to the infrequency of local festivals; moreover, since all the participants were not from the same area, collecting a large amount of data was difficult. Although earlier studies indicate that participation in festivals enhances individuals’ sense of community, they do not clarify whether the presence of dancing in festivals makes any difference in social bonds and the sense of community.

The current study addressed the aforementioned research gap as follows: A large-scale online survey assessed by three scales regarding sense of community and loneliness was conducted; further, the study examined whether the people who participate in local festivals involving dancing have a higher sense of community than those participating in festivals without dancing regardless of participation history or intention. This condition can clarify whether those who had participated in festivals and danced had a greater sense of community than those in other conditions. Research on whether festivals that involve individuals moving to music make an ongoing contribution to community maintenance, as well as enhancing individual social bonds, helps reassess the effects of dance from a long-term perspective and clarifies why festivals involving dance are held iteratively. This also serves as a link between the aforementioned experimental studies and real-world practices.

This study explored the psychological impact of the presence or absence of dancing at Japanese festivals. Approximately 600,000 festivals may be held in a year in Japan, of which around 300,000 are traditional festivals (Nikkei, Inc, 2015, August 23). Many of these are held in relatively small local communities. In many cases, they are held once a year or every few years. It has also been pointed out that many festivals are held in summer (Tanaka, 2024).

Specifically, Bon dance is one of the most popular dances in festivals in Japan (Gilhooly, 2009). Bon dance is an outdoor activity enjoyed by individuals of all ages and genders during the Obon season, which typically occurs between July and August. Originally emerging as a folk tradition tied to the Obon festival—a time when the spirits of the deceased are believed to return to this world to be honored—the dance has undergone a shift in meaning over time. Its religious connotations have diminished in recent years, and it has largely evolved into a community-oriented form of entertainment that welcomes participation from everyone. Furyu odori, the traditional Japanese dances in festivals including Bon dance have been registered as UNESCO Intangible Cultural Heritage. The Japanese Agency for Cultural Affairs has introduced them in a leaflet with photos of the dances (The Agency for Cultural Affairs, n.d.). Bon dance festivals are organized throughout Japan and have become widely recognized as casual events that people can enjoy in their regular clothing, even in urban areas. These festivals may also serve an important function in fostering a sense of community and bringing people closer together and a sense of sharing the same rhythm during dancing together that will provide a relief that we are living in harmony with each other (Oishi, 2020).

Accordingly, this study addresses the following hypotheses that (1) the participants of festivals involving dancing possess a higher sense of community than those who do not dance or who participate in festivals without dancing, (2) individuals’ high sense of community is not influenced by their previous festival participation habits, and (3) individuals’ high sense of community is not influenced by the intensity of their willingness to participate in festivals.

## 2 Methods

### 2.1 Participants

This study considered 1,768 participants (1,164 men, 603 women, and one other) with a mean age of 53.2 years (standard deviation, SD = 13.0). At the time of the study, the participants had been living in their current area of residence for at least 8 years, that is, residing in the same area for at least 5 years, excluding the COVID-19 period (during which many festivals were not conducted in Japan). Prior to conducting analyses, the participants who provided the same response (straight-line response) on more than one scale were excluded from the study. The correlation coefficient between the population of Japan’s 47 prefectures in 2023 and the number of participants in each prefecture in this study was 0.98 (*p* < 0.01). In other words, it can be said that the participants in this study are evenly distributed throughout Japan based on demographic data, without any regional bias.

### 2.2 Materials

Participants’ sense of community was assessed by incorporating the following three scales in the survey:

1. The Japanese version of the Brief Sense of Community Scale (BSCS; Yu et al., 2022) was used to measure the sense of community. This eight-item instrument comprises the following four factors: need fulfillment (the perception that members’ needs will be met by the community), membership (a sense of belonging or interpersonal relatedness), influence (the feeling that one is important, or able to make a difference, in a community and that the community is important to its members), and emotional connection (a sense of attachment or bonding based on the members’ shared history, place, or experience).
2. The short version of the Community Consciousness Scale (CCS; Ishimori et al., 2013) is a 12-item scale having the following four factors: solidarity (contribution to and active involvement in the community), self-determination (a sense of playing an active role in community improvement), attachment (attachment to and pride in the community), and dependency (letting others solve local problems).
3. The Japanese version of the UCLA Loneliness Scale Version 3 (UCLA-LS3-J; Arimoto and Tadaka, 2019) is a 10-item scale comprising a single factor. This scale was used to measure connectedness to others, unlike the two aforementioned scales.

### 2.3 Procedures

The survey was conducted with the help of a research company (GMO Research, Inc., Tokyo, Japan). Participants were presented with a description of the study and an informed consent form, and those who agreed to participate in the study proceeded to attend the survey. To minimize potential biases, the survey was conducted in December, outside the primary festival season in Japan, which usually takes place during summer and autumn, with a concentration of events like Bon dance in August.

The informed consent form stated that the survey would be analyzed anonymously, survey participation was not compulsory, the participants would not be disadvantaged in discontinuing or withdrawing from the survey, and their data would not be used for anything other than the study purpose. Only those who agreed to these conditions participated in the survey. This study was conducted in accordance with the Code of Ethics of the Japanese Psychological Association.

Initially, participants responded to a brief face sheet detailing their personal demographics. To eliminate differences among the participants based on their participation in the most recent local festival, the survey targeted those for whom the nearest festival was canceled from 2020 to 2022 because many festivals were canceled during this period to prevent the pandemic’s spread in Japan.

Subsequently, participants responded to the characteristics of local festivals (with or without dances with others) and their participation (whether or not they participated in 2023 and danced). The participants who attended more than one local festival a year were instructed to respond to the festival they were most familiar with. Participants also indicated their participation in festivals before COVID-19. Therefore, there are no duplicate participants when comparing averages in a single analysis below. For example, one participant will not be incorporated into the averages for both festivals with and without dancing. Statistical analysis was performed using IBM SPSS Statistics 24. ANOVA was performed on Type III SS.

## 3 Results

### 3.1 Months in which festivals are held

**Figure 1:**
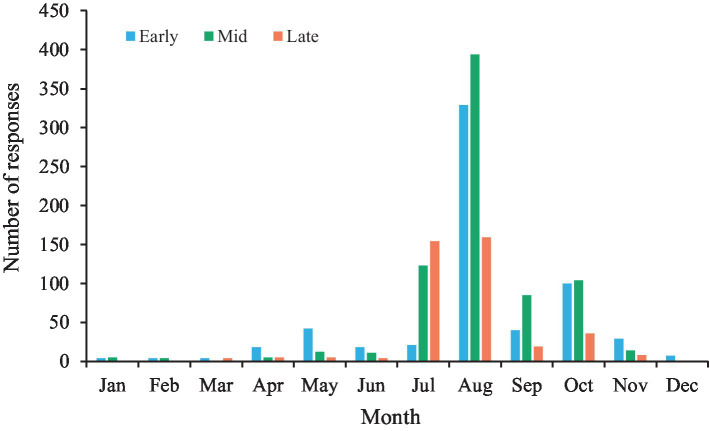
Month in which the festival was held for which responses were given.

August was the most common month in which festivals were held (49.9%); it was followed by July (16.9%) and October (13.6%). Details on the festival schedule are visualized in Figure 1. Hence, study results reflect this cultural aspect. The survey was conducted in December. Hence, many participants indicated in their survey responses that several months had passed since their last festival attendance.

### 3.2 Differences in sense of community based on types of festivals attended

To examine the relationship between how people participate in festivals and the level of their sense of community, scale scores were compared among three groups using analysis of variance (ANOVA): the participants who participated in festivals involving group dancing, those who participated in festivals involving dancing but did not dance themselves, and those who participated in festivals without dancing (Table 1).

**Table 1:** Mean ratings of scales and ANOVA results for participants’ sense of community by festival type.

Types of participation in festivals	Festivals involving dance		w/oD	*F*	*df*	*p*	*η_p_* ^2^	Multiple comparisons (Bonferroni)
	D	nD						
BSCS								
Need fulfillment	7.0	6.4	6.5	9.5	2, 700	0.000	0.026	D > nD**, D > w/oD**
Membership	7.3	6.5	6.9	9.9	2, 700	0.000	0.028	D > nD**, D > w/oD*
Influence	6.3	5.2	5.6	18.9	2, 700	0.000	0.051	D > nD**, D > w/oD**
Emotional connection	7.1	6.1	6.5	17.1	2, 700	0.000	0.047	D > nD**, D > w/oD**
CCS								
Solidarity	10.5	9.0	9.3	23.0	2, 700	0.000	0.062	D > nD**, D > w/oD**
Self-determination	11.1	11.1	10.5	3.2	2, 700	0.041	0.009	D > w/oD*
Attachment	10.3	9.8	9.6	6.1	2, 700	0.002	0.017	D > w/oD**
Dependency on others	8.6	9.1	9.1	4.3	2, 700	0.014	0.012	
**UCLA loneliness scale**	21.0	22.8	22.1	5.0	2, 700	0.007	0.014	D < nD*

D; Dancing, nD; not dancing, w/oD; without dancing. BSCS, Brief Sense of Community Scale; CCS, Community Consciousness Scale. **p < 0.01, *p < 0.05.

For each BSCS factor, the scores of the respondents who participated in a festival with group dancing were significantly higher than those of the respondents who did not dance or who participated in a festival without dancing. Further, for each CCS factor, except dependency on others factor, those who danced scored significantly higher than those who participated in a festival without dancing. Finally, for UCLA-LS3-J, those who danced scored significantly lower than those who did not dance at the festival. These results indicate that the sense of community among the participants who attended and danced at festivals was higher than that among the ones who attended festivals without dancing or who attended festivals with dancing but did not dance.

### 3.3 Sense of community when controlling for past participation

To control the effect of festival participation habits in the aforementioned results, we compared sense of community and loneliness only among participants who had attended the festival annually before COVID-19. Thus, an ANOVA was performed with festival type as the independent variable and sense of community scores as the dependent variable (Table 2). Results revealed that on the three BSCS subscales and two CCS subscales, the sense of community of those attending festivals with dancing was higher than that of those attending festivals without dancing. Loneliness was lower among the individuals who attended festivals with dancing than among those who participated in festivals without dancing. In other words, those who danced showed a higher sense of community, even among the participants having similar festival attendance habits, than those who did not.

**Table 2:** Mean ratings and ANOVA results for participants’ sense of community who attended festivals annually.

Types of participation in festivals	Festivals involving dance		w/oD	*F*	*df*	*p*	*η_p_* ^2^	Multiple comparisons (Bonferroni)
	D	nD						
BSCS								
Need fulfillment	7.3	7.0	6.6	3.4	2, 335	0.035	0.020	D > w/oD*
Membership	7.7	7.1	7.3	2.6	2, 335	0.075	0.015	
Influence	6.7	5.8	5.7	9.7	2, 335	0.000	0.055	D > nD*, D > w/oD**
Emotional connection	7.4	6.6	6.7	5.8	2, 335	0.003	0.034	D > nD*, D > w/oD*
CCS								
Solidarity	11.0	9.6	9.7	7.6	2, 335	0.001	0.043	D > nD*, D > w/oD**
Self-determination	11.3	11.4	10.8	1.4	2, 335	0.256	0.008	
Attachment	10.7	10.4	9.8	3.5	2, 335	0.030	0.021	D > w/oD*
Dependency on others	8.1	9.0	8.7	2.3	2, 335	0.098	0.014	
**UCLA loneliness scale**	20.1	21.5	22.5	4.2	2, 335	0.016	0.024	D < w/oD*

D; Dancing, nD; not dancing, w/oD; without dancing. BSCS, Brief Sense of Community Scale; CCS, Community Consciousness Scale. **p < 0.01, *p < 0.05.

### 3.4 Festival participation intent and sense of community

The sense of community of the respondents who participated in a festival was compared with that of the individuals who did not. In the analysis, the ones who did not participate were divided into two groups: those who wanted but were unable to participate and those who never intended to participate in the first place. This division helped identify the differences between respondents’ intention to attend and participation.

Results indicated that, for festivals with dancing, the ones who participated and danced scored the highest on all BSCS subscales (Table 3). The same trend was observed for CCS, with those who participated and danced having the highest sense of community. Further, loneliness was found to be the lowest among those who participated and danced. However, those who intended to but could not participate showed that either BSCS or CCS had similar results to those who participated but did not dance: no difference regarding loneliness was found among these two subcategories.

**Table 3:** Mean ratings and ANOVA results for respondents who participated in dancing festivals, wanted but could not participate, and had no intention to participate.

	AD	AnD	Unattended		*F*	*df*	*p*	*η_p_* ^2^	Multiple comparisons (Bonferroni)
			UI	UnI					
BSCS									
Need fulfillment	7.0	6.4	6.5	5.8	31.5	3, 1,042	0.000	0.083	AD>AnD, UnI**, AD>UI*, AnD > UnI*, UI > UnI**
Membership	7.3	6.5	6.7	5.8	41.7	3, 1,042	0.000	0.107	AD>AnD, UI, UnI**, AnD > UnI**, UI > UnI**
Influence	6.3	5.2	5.3	4.5	67.3	3, 1,042	0.000	0.162	AD>AnD, UI, UnI**, AnD > UnI**, UI > UnI**
Emotional connection	7.1	6.1	6.4	5.4	53.2	3, 1,042	0.000	0.133	AD>AnD, UI, UnI**, AnD > UnI*, UI > UnI**
CCS									
Solidarity	10.5	9.0	9.4	7.9	69.8	3, 1,042	0.000	0.167	AD>AnD, UI, UnI**, AnD > UnI**, UI > UnI**
Self-determination	11.1	11.1	10.9	10.4	5.8	3, 1,042	0.001	0.016	AD>UnI**, AnD > UnI*
Attachment	10.2	9.8	10.1	9.1	19.8	3, 1,042	0.000	0.054	AD>UnI**, AnD > UnI*, UI > UnI**
Dependency on others	8.5	9.1	8.7	9.5	10.7	3, 1,042	0.000	0.030	AD<UnI**, UI < UnI**
**UCLA loneliness scale**	21.0	22.8	21.7	24.4	23.3	3, 1,042	0.000	0.063	AD<AnD*, AD<UnI**, UI < UnI**

AD; Attended and Dancing, AnD; Attended, but not dancing, UI; Unattended, intended to participate, UnI; Unattended, no intention to participate, BSCS, Brief Sense of Community Scale; CCS, Community Consciousness Scale. **p < 0.01, *p < 0.05.

Similar tendencies were found for festivals without and those with dance; however, the number of pairs with significant differences between groups was fewer than that for festivals involving dance (Table 4). In particular, no significant differences were found between the participants who intended to attend but were unable to attend and those who did not intend to attend.

**Table 4:** Mean ratings and ANOVA for respondents in non-dancing festivals, those unable to participate, and those unwilling to participate.

	A	Unattended		*F*	*df*	*p*	*η_p_* ^2^	Multiple comparisons (Bonferroni)
		UI	UnI					
BSCS								
Need fulfillment	6.5	6.0	5.6	9.9	2, 495	0.000	0.039	A > UnI**
Membership	6.9	6.0	5.7	16.6	2, 495	0.000	0.063	A > UI**, A > UnI**
Influence	5.6	5.0	4.5	21.6	2, 495	0.000	0.080	A > UI**, A > UnI**, UI > UnI*
Emotional connection	6.5	5.6	5.2	19.7	2, 495	0.000	0.074	A > UI**, A > UnI**
CCS								
Solidarity	9.3	9.0	7.8	20.7	2, 495	0.000	0.077	A > UnI**, UI > UnI**
Self-determination	10.5	10.3	10.0	2.9	2, 495	0.058	0.011	
Attachment	9.6	9.3	8.8	5.9	2, 495	0.003	0.023	A > UnI**
Dependency on others	9.1	9.2	9.3	0.3	2, 495	0.743	0.001	
**UCLA loneliness scale**	22.1	23.7	24.0	5.2	2, 495	0.006	0.021	A < UnI**

A; Attended, UI; Unattended, intended to participate, UnI; Unattended, no intention to participate, BSCS, Brief Sense of Community Scale; CCS, Community Consciousness Scale. **p < 0.01, *p < 0.05.

Therefore, those who did not attend festivals, despite intending to attend, had a lower sense of community than those who did. This tendency was particularly prominent among festivals involving dance.

## 4 Discussion

This study investigated how dancing at local festivals affects individuals’ sense of community. A survey that was conducted a few months after a community festival revealed the following findings: (1) The participants who attended a festival involving dancing and actually physically participated in dancing had a higher sense of community and lower loneliness than those who did not dance or who attended the festival without dancing. (2) These tendencies were not influenced by previous festival attendance habits. (3) These tendencies were not related to individuals’ willingness to attend festivals. Accordingly, these results suggest that dancing at festivals can promote a sense of community since it is not significantly influenced by individuals’ habits or willingness to participate.

### 4.1 Relationship between dance at festivals and their sense of community

The participants who attended festivals with dancing and danced had a higher sense of community and lower level of loneliness than those who did not dance or those who participated in festivals without dancing. Prior qualitative research has shown that participating in local festivals and dancing with others at local events are associated with a sense of community (Kino, 2016) and intergenerational solidarity (Argan et al., 2021). On the other hand, it has not been quantitatively clarified to what extent there is actually a difference in sense of community and loneliness between dancing and not dancing in those festivals. This study found that dancing with others at daily festivals in real-life settings is associated with increased sense of community and decreased loneliness. These results extend the findings of earlier studies in laboratory settings because it focuses on the social function of dancing at real-world festivals, which has been practiced since ancient times, and highlights the significance of dance. Further, the results add a new perspective to the findings of a series of studies that indicate how dance deepens social bonds (Dunbar, 2011; Savage et al., 2020; Trehub et al., 2015) by examining the presence or absence of dance. Furthermore, the connection between dance and a sense of community in the real world sheds light on the social meaning of the fact that the urge to move one’s body to music is observed across cultures (Etani et al., 2024; Kawase, 2024; Kawase and Eguchi, 2010).

Additionally, the current results are consistent with the findings of experimental research on social bonding with those who move synchronously with others to music (Dunbar, 2011; Fitch, 2015; Savage et al., 2020; Tarr et al., 2016) and on prosocial behaviors that are exhibited by individuals (from 14-month-olds to high school students) immediately after they move to music (Cirelli et al., 2014; Tarr et al., 2015). Many of these studies regard experimental synchronous movement as a controlled, cooperative dance, and focus on the role of dance in the formation of society in human evolution. Furthermore, the present results are supported by the earlier findings that moving together in accordance with music, or simply moving together with other people promotes sociability (Cirelli et al., 2014; Kawase et al., 2018; Kirschner and Tomasello, 2010) and social bonding (Stupacher et al., 2017) prosocial behavior (Carpenter et al., 2013; Keller et al., 2014), altruistic behavior (Valdesolo and DeSteno, 2011), interpersonal likeability (Hove and Risen, 2009), rapport (Lakens and Stel, 2011), trust (Launay et al., 2013), and bonding with others (Tarr et al., 2016). The triggers for such prosocial aspects were reportedly the effects of transient physiological responses, such as the release of endorphins (e.g., Tarr et al., 2016). Brain mechanisms could also explain these social bonding effects through increased neural synchrony and enhanced interpersonal coordination (Basso et al., 2020).

Contrastingly, the current results indicate the long-lasting effects of dancing to music because this study was conducted several months after a festival that involved dancing. Community festivals and ceremonies occur only a limited number of times (usually once) a year. Although it does not clarify whether moving together to music can generate lasting social bonds, this study suggests that moving to music at a festival but only a limited number of times contributes to not only the enhancement of individual social bonds but also the ongoing maintenance of the community. Further, the results clarify that the inclusion of synchronous dancing in festivals is associated with a high sense of community. A large body of literature indicates that participation in festivals increases individuals’ sense of community (Arcodia and Whitford, 2006), and accordingly, festivals promote community awareness (Hassanli et al., 2021), building (Mair and Duffy, 2020), and maintenance (Black, 2016) and enhance social sustainability. The current results, which are characterized by a high sense of community and a low level of loneliness, are consistent with the aforementioned findings.

However, the results suggest that the sense of community and level of loneliness differ according to the festival type, that is, the presence or absence of group dancing. Given that group dancing or moving together enhanced prosociality of participants toward those who did not dance with them (Reddish et al., 2016; Tarr et al., 2015), dancing together can affect prosociality not only towards those who danced together but also toward a broader range of members of the surrounding community. Therefore, it is possible that dancing and moving together at festivals increased closeness not only toward those who were present but also toward people in the community, even though they were not dancing together, and strengthened sense of community of festivals attendees who danced together. Further investigation into dancing at festivals should explore this possibility.

Furthermore, this study indicated that dancing may enable the formation and maintenance of social bonds, even in large community groups. Weinstein et al. (2016) showed that, in choral singing, even large groups that are less familiar with each other generate social bonds during collective singing to the same extent as in intimate small groups. Thus, social bonds are generated even in festivals where large numbers of community members participate. As for synchronous group dancing, and in line with the current results, it is suggested that social bonding can be appropriately extended to match the increase in group size, which makes dancing an effective method to connect with many people simultaneously.

The present results also suggest that the sense of community and feelings of loneliness are also influenced by the benefits of the dance itself. Dance exerts many positive effects on the mind and body (Fong Yan et al., 2024; Koch et al., 2014; McCrary et al., 2021; Vander Elst et al., 2023) in terms of improving physical function, cognitive function, and rehabilitation of neurological disorders and reduction of mental stress, depression, and anxiety. Furthermore, group dance activities promote social interaction and contribute toward reducing feelings of isolation (McCrary et al., 2021). Given these benefits of dance, festivals that involve dance could help to improve mental and physical stability and build social bonds while enhancing a sense of community.

### 4.2 Relationships among individuals’ festival attendance habits, intention to attend festivals, and sense of community

The observation of the tendency for those who attend and dance in festivals to have a higher sense of community and a lower loneliness level, but being unrelated to their past festival attendance habits, can perhaps be interpreted by that those who dance possess a higher sense of community than others. However, in this study, those with a high frequency of past (pre-COVID-19) festival participation and those with high intentions to participate in festivals did not possess a higher sense of community than those who danced during the present year of observation. In other words, even if individuals intend to attend a festival, their sense of community does not become high if they do not dance at the festival. A link was observed between motivation, the quality of the experience, and satisfaction with participation in the Turkish dance festival (Argan et al., 2021). On the other hand, the results of this study show that, even if people had the intention to participate, those who did not participate in the festival showed a lower sense of community, indicating the importance of participating, dancing, and motivation. Furthermore, dancing at festivals may also provide participants with satisfaction. This virtuous cycle may increase their motivation to participate in the following year. Nonetheless, given the existence of very few studies on the impact of dance on festivals, the current study provided additional significant support for the impact of dance on festivals from a social perspective. Accordingly, it is important to move and share places and times to receive the benefits of festivals involving group dancing.

Interestingly, the effects of festival participation may not last for years. The sense of community of those who participated in the festival every year before COVID-19 but did not participate thereafter was not higher than that of the ones who danced after the pandemic. In other words, the effects of dancing at festivals may fade after a few years. This study was conducted toward the end of the COVID-19 pandemic, at a time when the restrictions that had been in place for approximately 3 years were relaxed and festivals resumed. Therefore, even if people had been attending festivals and dancing before COVID-19, the effect of attendance on their sense of community and loneliness would have diminished if they had been interrupted for some time. This means that the effect of festival participation can be considered to continue on a monthly, not yearly, basis. Consequently, one reason why some individuals continue participating in festivals may be the ongoing enhancement of their sense of community.

## 5 Limitations and future research

This study did not specify the long-term effects of festivals lasting multiple years. Hence, further investigation into the effects of participation in festivals with dancing is necessary. In general, festivals are held once a year or every few years. Therefore, it is necessary to focus on the sustainability of maintaining or attenuating the effects of participation in festivals. In addition, a method for randomly selecting participants is necessary. By randomly assigning festival participants to different conditions, the festival’s effects on communal awareness can be measured under controlled conditions. Long-term follow-up surveys may also be effective. The simplest and most ideal method would be to randomly assign local participants to either “dance” or “not dance” and then continuously measure their sense of community. Furthermore, it is useful to understand the impact of differences in festival dance type and participation style on sense of community. For example, investigations into ethnic dances worldwide and casual brief participation in festival dances could provide valuable insights.

Experiences related to individual dance should be considered in future studies, since this study has not collected data regarding individual dance experience, for instance, dance lessons. To shed more light on the topic, international and cultural comparisons must be performed. In Japan, many different festivals are held; however, the forms of festivals and the attitudes of participants differ across cultures.

## 6 Conclusion

This study clarified that those who attended and danced with others at festivals involving dancing had a greater sense of community and lower loneliness than those who did not dance or those who attended festivals without dancing. These tendencies were unrelated to past participation habits or a high level of willingness to participate, indicating the importance of continuously participating in and dancing at festivals. Results provided new insights into the beneficial effects of moving the body in tune with music, which is a universally observed behavior, and its social significance. The famous Japanese saying *Odoranya son* (It’s a loss if you do not dance) indicates that dancing can foster social bonds and alleviate loneliness while in present communities, reduced social bonds and intense feelings of loneliness are significant social problems (Toepoel, 2013). Since ancient times, local festivals have helped foster bonds between community members. This study can help reevaluate the roles of dance in festivals and shed light on the reasons for different human cultural practices.

For practical applications, this study highlights a simple solution to strengthen individuals’ sense of community. By simply participating in local festivals involving dances, for example, Bon dance, individuals can increase their sense of belonging and social bonding. Sharing dance and music at festivals may help people experiencing verbal communication difficulties to socially bond with others since dance, in general, requires no words. Furthermore, a quantitative view of social bonding can help reassess the social value of local festivals.

## Data Availability

The raw data supporting the conclusions of this article can be available from the corresponding author on reasonable request.
